# Retraction Note: Circular RNA hsa_circ_0055538 regulates the malignant biological behavior of oral squamous cell carcinoma through the p53/Bcl-2/caspase signaling pathway

**DOI:** 10.1186/s12967-023-04147-x

**Published:** 2023-05-08

**Authors:** Wen Su, Shuai Sun, Feng Wang, Yuehong Shen, Hongyu Yang

**Affiliations:** 1 Clinical School, Peking University Shenzhen Hospital, Anhui Medical University, Shenzhen, 518036 Guangdong China; 2 Department of Oral and Maxillofacial Surgery, Peking University Shenzhen Hospital, No. 1120 Lianhua Road, Shenzhen, 518036 Guangdong China

**Retraction: J Transl Med (2019) 17:76** **https://doi.org/10.1186/s12967-019-1830-6**

The Editor-in-Chief has retracted this article at the corresponding author's request. After publication, concerns were raised regarding the images presented in the figures. Specifically:

In Fig. 1d, the Control and Test images appear to originate from the same sample.

Fig. 2d Control appears highly similar to Fig. 2b circRNA overexpression group in the authors' other article that was under consideration within a similar time frame [1].

In Fig. 4a, the Control and Test 0h images appear to overlap.

In Figs. 4b and 6b, the 0h images appear to contain overlapping areas.

In Fig. 4c, there appears to be an overlapping area between the SSC9 Control and Test images.

In Fig. 7d, based on the shapes of the bands, it appears that the caspase-3 blot is shifted by one lane, so the group labels don't seem to match the samples.

The authors have checked the data underlying this article and stated that incorrect images were used. The authors and the Editor-in-Chief therefore no longer have confidence in the presented data.

Wen Su, Feng Wang, Yuehong Shen and Hongyu Yang agree to this retraction. The Publisher has not been able to obtain a current email address for Shuai Sun.
